# Nonlinear Optical Properties of Zirconium Diselenide and Its Ultra-Fast Modulator Application

**DOI:** 10.3390/nano9101419

**Published:** 2019-10-04

**Authors:** Mengxiao Wang, Yue Zheng, Linguang Guo, Xiaohan Chen, Huanian Zhang, Dengwang Li

**Affiliations:** 1 Shandong Province Key Laboratory of Medical Physics and Image Processing Technology, School of Physics and Electronics, Shandong Normal University, Jinan 250014, China; 2016020640@stu.sdnu.edu.cn (M.W.); yuezheng_9412@163.com (Y.Z.); linguang_guo@163.com (L.G.); 2 School of Information Science and Engineering, and Shandong Provincial Key Laboratory of Laser Technology and Application, Shandong University, Jinan 250014, China; cxh@sdu.edu.cn; 3 School of Physics and Optoelectronic Engineering, Shandong University of Technology, Zibo 255049, China

**Keywords:** Zirconium diselenide (ZrSe_2_), nonlinear absorption properties, saturable absorber (SA), Er-doped fiber laser, passively mode-locked fiber laser

## Abstract

Recently, two-dimensional (2D) materials have been widely studied by researchers due to their exceptional 2D structure and excellent optical characteristics. As one of the typically-layered 2D transition metal dichalcogenide (TMD) semiconductors from group IVB with a bandgap value of 0.9–1.2 eV (bulk to monolayer), the characteristics of zirconium diselenide (ZrSe_2_) have already been extensively investigated in many fields. However, the nonlinear absorption properties of ZrSe_2_ in ultra-fast lasers have not been previously demonstrated. In this work, we measured various parameters in order to investigate the characteristics of the nonlinear saturable absorption of ZrSe_2_. A ZrSe_2_–polyvinyl alcohol (PVA) film was successfully prepared, which was employed as a saturable absorber (SA) to demonstrate, for the first time, an erbium (Er)-doped passive mode-locking fiber laser with a ring cavity. The saturation intensity of the ZrSe_2_–PVA film-type SA is 12.72 MW/cm^2^, while its modulation depth is 2.3%. The stable soliton state with a maximum output power of 11.37 mW and a narrowest monopulse duration of 12.5 ps at a repetition frequency of 21.22 MHz was detected. The experimental results conclusively proved that ZrSe_2_, with its suitable bandgap value and excellent nonlinear absorption properties, as well as its high damage threshold, should have extensive potential applications within the field of ultra-fast pulse lasers.

## 1. Introduction

Mode-locked fiber lasers have been arousing the extensive interest of many researchers due to their value in a variety of applications in the fields of basic science, fiber telecommunications, industrial materials processing, precise optical metrology, biomedical imaging, and others [1,2,3,4,5,6,7,8,9,10]. Currently, there are normally two strategies, actively mode-locked and passively mode-locked technologies, that are employed to realize the mode-locked operation in a fiber laser. In order to achieve the actively mode-locked scheme, an acoustic-optic or electro-optic modulator is usually essential, which makes the laser complex so that it becomes difficult to achieve miniaturization. Compared to the actively mode-locked method, the passively mode-locked technique has more attractive advantages, such as no requirement for modulators, low cost, a compact structure, good stability, etc. Thus, it has been desired for a variety of studies and applications [9,11]. As is known, the nonlinear optical element, called a saturable absorber (SA), is one of the key devices for achieving various ultra-short pulse trains and enhancing the stability of the environment in a passively mode-locking fiber laser [4,12]. Therefore, the study of novel and high performance SAs has always been the focus of ultrafast laser research.

Over the past decade, extensive investigations on various two-dimensional (2D) materials, which were employed as SAs because of their excellent nonlinear saturable absorption characteristics, have actually facilitated the rapid development of passively mode-locking fiber lasers [8]. As we all know, the discovery of graphene plays a crucial role in the exploration of novel 2D SAs [13,14,15,16,17,18,19,20,21,22]. Graphene exhibits excellent ultra-broadband absorption characteristics as a SA due to its unique bandgap structure [23,24,25,26,27]. However, two main disadvantages of graphene, the weak modulation depth of about 1.3% per layer and the difficulty in creating an optical bandgap, restricted its further in-depth application in passive mode-locking fiber lasers [4,28]. Since then, after graphene has been widely studied, different kinds of graphene-like 2D materials, including topological insulators (TIs) [29,30,31,32,33,34,35,36], transition metal dichalcogenides (TMDs) [37,38,39,40,41], and black phosphorus (BP) [42,43,44,45,46,47], have been widely used as SAs for investigating the characteristics of passively mode-locking fiber lasers. Significant progress has been made in the study of the SA base on these layer-structured 2D materials recently. Based on this, exploration of the new types of SAs with high damage thresholds, broadband absorption and ultrafast recovery time is still urgent due to the ever-increasing demand for mode-locked fiber lasers.

Recently, 2D semiconducting TMDs from group IVB, with the chemical formula MX_2_ (M = Ti, Zr, Hf; X = S, Se, Te) have been extensively investigated because of their suitable wide-range bandgaps, their superior physical, electronic, magnetic, electrochemical, and photoelectrochemical (PEC) properties, and so on [48,49,50,51,52,53]. It has been reported that a stable TMD monolayer from group IVB is formed by one plane of a transition metal (M) and two slabs of chalcogen atoms (X), called a X-M-X-type sandwich, where M and X atoms are tightly bonded together by covalent bonding, and the layers are stacked together via weak van der Waals interactions [54]. As one of the typically-layered 2D TMD semiconductors from group IVB, zirconium diselenide (ZrSe_2_) has garnered special interest from researchers recently. In 2017, the unique layer number dependence of the bandgap value for ZrSe_2_ was demonstrated by Michal et al., where a ZrSe_2_ monolayer has an indirect bandgap value of 1.2 eV, and the bandgap value of a ZrSe_2_ multilayer is 0.9 eV, which facilitates the low-voltage operation of nanoscale transistors [55]. Zhang et al. studied the room temperature mobility of 14 different 2D semiconductors, and proved that ZrSe_2_ had a remarkably high photon-limited mobility of above 2300 cm^2^ Vs^−1^ at room temperature, which will be one of the important factors for the future development of high-performance nanoelectronics and optoelectronics [48,56]. In 2018, based on the first-principles calculation within the framework of density functional theory, Zhao et al. modulated the electronic and magnetic properties of single layer ZrSe_2_ by doping. Their finding suggested that ZrSe_2_ is a promising candidate for promoting progress in spintronic devices [50]. Furthermore, Jiang indicated that ZrSe_2_ can be considered to be a potential candidate for third-generation solar cells owing to its bandgaps within the range of the visible and infrared [53]. Additionally, the thermoelectronic properties and electrochemical characteristics of ZrSe_2_ have also been investigated by some researchers, who found that single layer ZrSe_2_ has more excellent thermoelectronic properties than that of MoS_2_ and MoSe_2_ (the 2D semiconducting TMDs from group VIB) [50,51,57]. However, to the best of our knowledge, no previous studies have investigated the nonlinear absorption properties of ZrSe_2_ in ultra-fast lasers. Owing to its comparable properties with previously reported TMDs, ZrSe_2_ is expected to exhibit equally excellent saturable absorption properties.

In this work, a ZrSe_2_–polyvinyl alcohol (PVA) film was successfully prepared, which was employed as a SA to demonstrate a passively mode-locking erbium (Er)-doped fiber laser for the first time. Additionally, the appearance and nonlinear optical absorption characteristics of the prepared ZrSe_2_–PVA film were also investigated, and the saturation intensity and modulation depth were 12.72 MW/cm^2^ and 2.3%, respectively. A stable soliton state with a maximum output power of 11.37 mW and a narrowest monopulse duration of 12.5 ps at a repetition frequency of 21.22 MHz was detected. Our experimental results proved that, compared with the reported TMDs before, ZrSe_2_ has parallel optical absorption characteristics and equally excellent performance, which shows that ZrSe_2_ can extensively promote the development of ultrafast pulse fiber lasers.

## 2. Preparation and Characterization of ZrSe_2_-Based SAs

### 2.1. Preparation Process of ZrSe_2_ SAs

**Figure 1:**
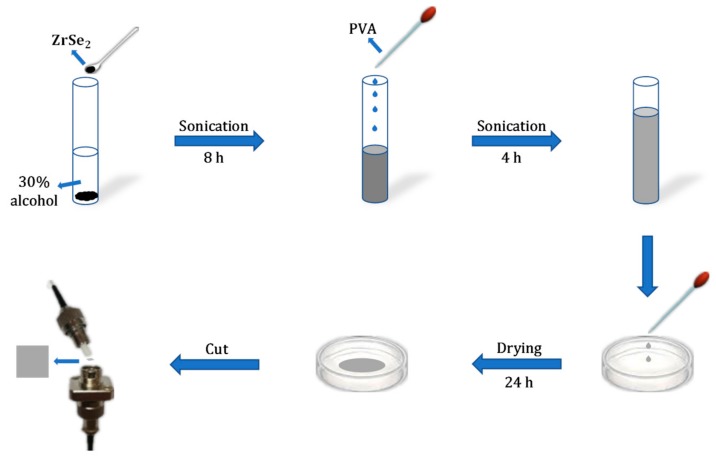
The preparation process of the ZrSe_2_-PVA film-type SA.

Figure 1 displays the process of preparing the ZrSe_2_–PVA film-type SA. As is shown in the figure, a common method called liquid-phase exfoliation was applied in our experiment for preparing the ZrSe_2_ dispersion solution, as well as the ZrSe_2_–PVA dispersion solution, while the formation of the ZrSe_2_–PVA film-type SA utilized the spin coating method. For a start, 0.1 g ZrSe_2_ nanocrystals (NCs) were mixed with 30 mL 30% alcohol. The mixture was placed in a high-power ultrasonic cleaner for 8 h and then centrifuged at a speed of 2000 rpm for 30 minutes in order to prepare the ZrSe_2_ homogeneous mixture. Hence, the ZrSe_2_ solution was acquired. Next, a 5 wt.% PVA solution was added into the ZrSe_2_ homogeneous mixture at a volume ratio of 1:1. The homogeneous ZrSe_2_–PVA dispersion solution was obtained after placing the mixture in the working ultrasonic cleaner for 4 h. In our work, PVA was selected as a matrix instead of other commercially-available materials due to its excellent properties of long time stability, low cost, high damage threshold, easy film formation, and so on. After that, in order to obtain a thin film, 150 μL ZrSe_2_–PVA homogeneous mixture was spin coated onto a culture dish and dried in the drying oven at 30 °C for 24 h. Finally, a 1 × 1 mm^2^ square was stripped from the thin film and placed between the two facets of the fiber jumper and connected with a connector for making SAs. In this way, the ZrSe_2_–PVA film-type SA could be prepared successfully.

### 2.2. Characterization Results of ZrSe_2_ SAs

**Figure 2:**
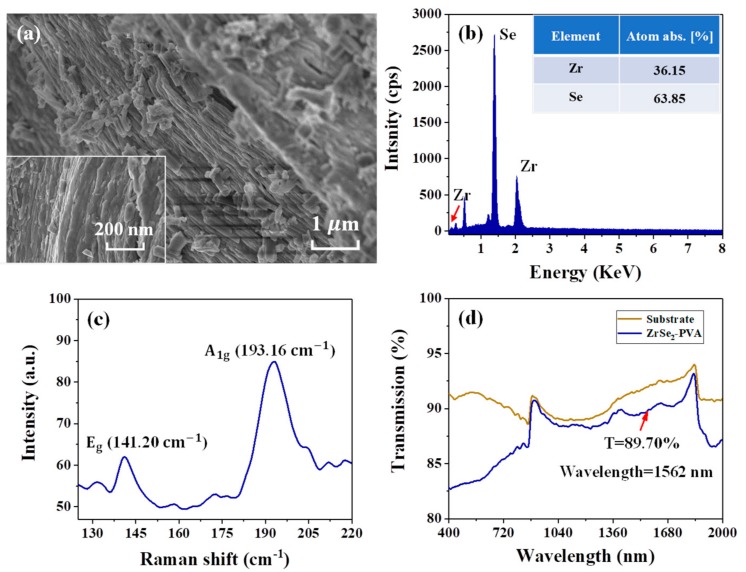
(**a**) Scanning electron microscopy (SEM) image of the ZrSe_2_ nanocrystals (NCs) at a resolution of 1 μm. Inset shows the SEM image at a resolution of 200 nm. (**b**) Energy dispersive spectrometer (EDS) spectrum of the ZrSe_2_ NCs. (**c**) Raman spectrum of the ZrSe_2_ power. (**d**) Linear transmission curve of the ZrSe_2_–PVA film and the substrate as a function of the wavelength.

In order to investigate the characteristics of the ZrSe_2_ NCs, we used a scanning electron microscopy (SEM, Sigma 500, ZEISS, Zeiss, Jena, Germany), an energy dispersive spectrometer (EDS, QUANTAX EDS, Bruker, Karlsruhe, Germany) and a Raman spectrometer (Horiba HR Evolution, Kyoto, Japan). It is well known that the surface morphology characteristics of the samples used in our experiment are important for analyzing the performance of the SA. Thus, based on SEM, the SEM image of the ZrSe_2_ NCs at a resolution of 1 μm is shown in Figure 2a; moreover, the inset in Figure 2a illustrates a zoomed-in SEM image at a resolution of 200 nm. As is depicted, the used sample exhibits an obvious typical layered structure, which indicates that a monolayer or a few layers of ZrSe_2_ NCs will be obtained after using liquid-phase exfoliation technology. For measuring the quantitative elemental composition of the ZrSe_2_ NCs, EDS was applied for characterizing the microstructure. Figure 2b depicts the chemical composition of our sample. As is shown, the typical peaks attributed to Zr and Se can be easily observed; meanwhile, the atomic ratio of Zr and Se is 36.15:63.85, which is close to 1:2. The structural characteristics of the ZrSe_2_ NCs, which were excited by a 532 nm laser, were also investigated using Raman spectroscopy. It can be seen from Figure 2c, the Raman spectrum with two characteristic peaks was detected in the range of 125–220 cm^−1^, where the two active modes are A_1g_ and E_g_ modes with photon frequencies of 193.16 and 141.20 cm^−1^, which corresponds well with previous work [58]. These results suggest that the pure ZrSe_2_ NCs were prepared successfully. In addition, in order to characterize the absorption characteristic of the ZrSe_2_–PVA film, the linear transmission of the ZrSe_2_–PVA film and the substrate as a function of the wavelength were investigated using a ultraviolet/visible/near infrared (UV/vis/NIR) spectrophotometer (Hitachi U-4100, Tokyo, Japan). It is obvious that the linear transmittance of the ZrSe_2_–PVA film increased with the increase of the optical wavelength, as shown in Figure 2d, while the transmission was measured as 89.70% at the wavelength of 1562 nm. Furthermore, as is seen from Figure 2d, the ZrSe_2_–PVA film has a broad range of absorption, which extends from visible light to near infrared (NIR) light, indicating that the ZrSe_2_–PVA film can be considered as promising for the development of photonics devices in the visible or NIR region.

**Figure 3:**
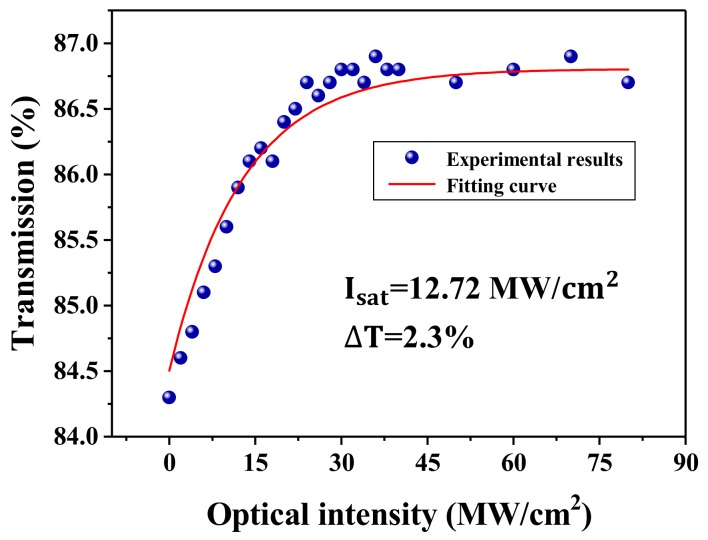
The nonlinear absorption characteristics of the ZrSe_2_–PVA film.

Additionally, the nonlinear saturable absorption characteristics of the ZrSe_2_–PVA film were also studied by using transmission technology related to power. A home-manufactured nonlinear polarization rotating Er-doped mode-locking fiber laser with a pulse duration of 560 fs under a center wavelength of 1580 nm, as well as a repetition frequency of 33.6 MHz, was used as the pump source. The experimental results, which were recorded by two power meters, can be seen in Figure 3. Furthermore, the following conventional equation can be employed for fitting the obtained experimental data [59]:

(1) $$T\left(I\right)=1-\Delta T\times \exp\left(-I/I_{sat}\right)-T_{ns}$$

where $T\left(I\right)$ is the transmission rate, I is the input intensity of the laser, ΔT represents the modulation depth, $I_{sat}$ is the saturation intensity, and $T_{ns}$ is the non-saturable absorbance. Figure 3 also depicts the fitting curve of the experimental results. As is shown, the saturation intensity of the ZrSe_2_–PVA film is 12.72 MW/cm^2^, while the modulation depth is 2.3%, indicating that the prepared ZrSe_2_–PVA film can be used as an excellent SA.

## 3. Experimental Setup

**Figure 4:**
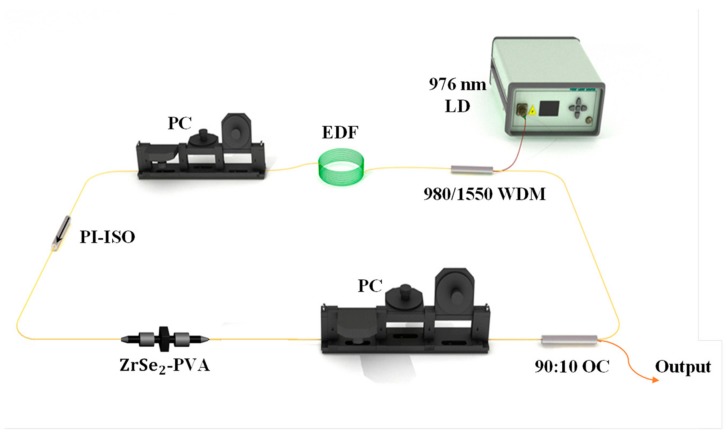
A schematic diagram of the experiment for the ZrSe_2_-based passively mode-locking Er-doped fiber laser.

Figure 4 shows a schematic diagram of our experiment. In our work, the pump source, which was a 976 nm laser diode (LD), was employed for generating a 980 nm laser. A ring cavity laser, including a wavelength division multiplexer (WDM) (980/1550), a Er-doped fiber (Er-80,8/125), a polarization-independent isolator (PI-ISO), a ZrSe_2_–PVA film-type SA, two polarization controllers (PCs), and a 90:10 output coupler (OC), was employed for investigating the performance of the ZrSe_2_–PVA film. The Er-doped fiber served as the laser gain medium, which had a length of 37 cm and a dispersion parameter of approximately 15.4 ps/(nm·km), while the dispersion parameter of the single-mode fiber used in this study was about 17.7 ps/(nm·km). The PCs were employed for changing the polarization state, and the PI-ISO was used for ensuring unidirectional delivery of light through the cavity. The ZrSe_2_–PVA film-type SA was placed between the PI-ISO and one of the PCs. The 90% OC port was considered as one part of the ring cavity, while the 10% port was used to export the laser. Additionally, it is an indisputable fact that, in an all-anomalous-dispersion regime, the generation of various solitons is attributed to the dynamic equilibrium between the anomalous dispersion, the fiber nonlinear effects, and the total cavity gain and loss. In our experiment, the mode-locking operation was detected when the length of the total cavity reached 9.67 m by changing the state of the PCs, as well as the amplitude of the pump power. Thus, the intracavity net dispersion was calculated to be about −0.2213 ps^2^, with a cavity length of 9.67 m. Additionally, a fast-speed photodetector (3GHz), a digital oscilloscope (DPO3054), an optical spectrum analyzer (AQ6317B, Yokogawa, Tokyo, Japan), a radio-frequency (RF) spectrum analyzer (R&S FPC1000, Jena Germany) and a power meter (PM100D-S122C, Thorlabs, New Jersey, American) were employed to record the output characteristics of the fiber laser.

## 4. Results and Discussion

In order to investigate the importance of the ZrSe_2_–PVA film-type SA in the fiber laser we designed, we firstly removed the SA from the ring cavity before carrying out this experiment. Only the continuous-wave state was recorded when the pump power and the PCs were adjusted. Additionally, after the ZrSe_2_–PVA SA was inserted into the ring cavity, the stable mode-locking state was found by carefully changing the PCs under the pump power of 351 mW, which suggested that the pulse generations could be attributed to the nonlinear absorption of the SA. Obviously, the threshold power detected was slightly higher, which was generated mostly by the high insertion loss of the SA. The Q-switched operation could sometimes be recorded when the cavity length of the fiber laser was set to be short. However, no Q-switched operation occurred when the PCs and pump power were adjusted in this work, which indicated that the SA may exhibit a low modulation depth and saturation intensity [11].

**Figure 5:**
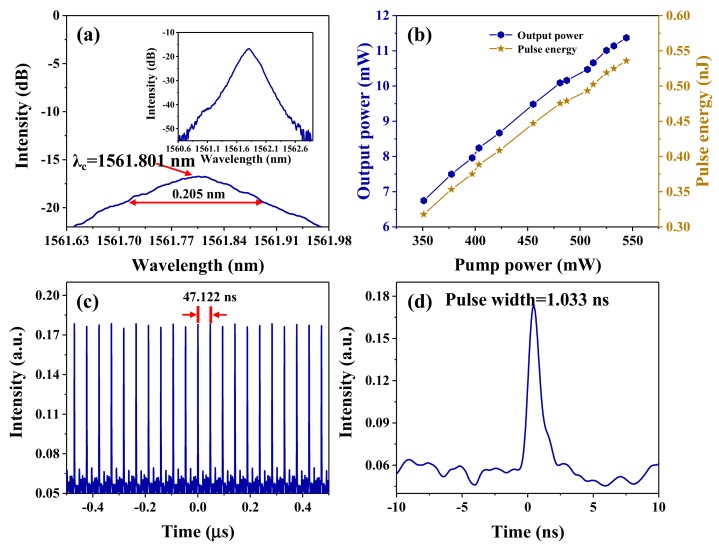
(**a**) The output optical spectrum (inset: the emission optical spectrum with a 2 nm span). (**b**) The output power and the single pulse energy versus the pump power. (**c**) The typical pulse train of the mode-locking operation. (**d**) The zoomed-in monopulse profile of the mode-locking operation.

In this section, we discuss the characteristics of the generated mode-locking operation with a pump power of 544.1 mW in Er-doped fiber lasers. A typical optical spectrum occurred when the center wavelength was located at 1561.801 nm, which was recorded by the optical spectrum analyzer at a resolution of 0.02 nm, as shown in Figure 5a. It can be seen that the 3 dB bandwidth was only 0.205 nm, and, in comparison to previous works [39,41,42], the recorded 3 dB width was narrower, which can be explained by the shaping of the spectrum by the filtering effect of the unidirectional ring (UR) [60,61,62,63]. Comparing the bandgap value of ZrSe_2_ (0.9–1.2 eV), the operating photon energy (0.79 eV) was lower, which indicates that the generation of the soliton was due to sub-bandgap absorption. Recently, the effects of sub-bandgap absorption phenomena on the passive mode-locking operation have been reported widely [64,65,66,67]. In a finite system, the energy levels in the bandgap deriving from the edge-state contribute to the sub-bandgap absorption under a low photon energy [59,66,67,68]. Therefore, we can conclude that the sub-bandgap absorption of ZrSe_2_ seen in our study could also have arisen from its edge-state absorption. Additionally, the net dispersion value of the designed ring-cavity was −0.2213 ps^2^. It is generally known that obvious Kelly sidebands should be detected in the output optical spectra in an all-anomalous-dispersion regime. However, the optical spectrum was smooth and no Kelly sidebands were recorded as shown in the inset of Figure 5a. As is known, elimination of the Kelly sideband will significantly improve the quality of the pulses [69,70,71,72], which explains the improvement seen in our results. In Figure 5b, the correlation between the output powers and pump powers are depicted. With the increase in pump power from the mode-locked threshold to the full pump of 544.1 mW, the stable mode-locking operation could always be detected. Meanwhile, when the pump power was maintained for 15 min at 544.1 mW, and then reduced to less than 500 mW, the mode-locked phenomenon could still be recorded, which means that the SA has a high damage threshold, which was greater than 544.1 mW. Moreover, the optical conversion efficiency was calculated to be 2.09% at the maximum output power of 11.37 mW. As described above, the output power and optical-to-optical conversion efficiency both had low values, which were also mostly attributed to the high insertion loss of the SA; the insert loss of the SA was calculated to be about 0.5 dB from the data of Figure 2d. Therefore, it was necessary for us to reduce the total insertion loss in order to obtain a low threshold and high optical conversion efficiency, and this will be investigated in one of our future studies. We detected the typical pulse train of the mode-locking operation by the photodetector combined with the digital oscilloscope, which is displayed in Figure 5c, where the top height of the mode-locking pulse train is consistent, without obvious fluctuation. As can be seen from Figure 5c, the time interval from pulse to pulse was 47.122 ns with a repetition frequency of 21.22 MHz, matching well with the round-trip time of the ring laser cavity, which indicated that the output pulses were attributed to the mode-locked operation. Thus, as depicted in Figure 5b, the single pulse energy increased from 0.32 to 0.54 nJ according to the output powers and the repetition frequency of 21.22 MHz. Figure 5d illustrates a zoomed-in monopulse sharp involved in the mode-locking pulse train with a monopulse duration of 1.022 ns. It is worth mentioning that the single pulse duration recorded here cannot reflect the real pulse width of the ultra-fast pulse because of the slow response times of the photodetector and the digital oscilloscope. Thus, based on the theory of mode-locked soliton, we assumed that the obtained laser pulse shape was the perfect Sech type. The theoretical pulse duration Δτ can be calculated based on the following formula, called the time-bandwidth product (TBP):

(2) $$TBP=\Delta \tau \times \left(\frac{c}{\lambda_{c}{}^{2}}\;\times \;\Delta \lambda \right)$$

where *c* represents the light velocity (2.9979 × 10^8^ m/s), $\lambda_{c}$ is the center wavelength, and Δλ is the spectrum width (full width at half maxima, FWHM). Thus, based on the above data, the calculated pulse duration Δτ is approximately 12.5 ps. However, due to the narrow 3 dB spectrum width, the pulse width was wider than previous works [34,35,37,39]. In our experiment, we also recorded other soliton operations; however, the solitons detected could not exhibit a stable state. Therefore, in our next work, in order to investigate more mode-locking phenomena, we will try to optimize the parameters of the SA, such as the modulation depth and saturation intensity, or change the dispersion of the resonant cavity by adjusting the length of the Er-doped and single-mode fiber.

**Figure 6:**
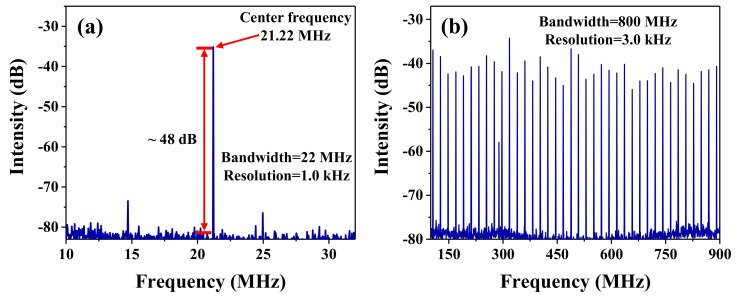
(**a**) The RF output spectrum of the mode-locking laser with the fundamental frequency of 21.22 MHz. (**b**) The RF spectra with a band width of a 800 MHz range.

It is well known that the stability can be considered one of the crucial factors in determining whether the mode-locking fiber laser can be used for practical applications. For testing the stability of the mode-locking fiber laser based on the ZrSe_2_–PVA film-type SA, the RF spectra were measured using the RF spectrum analyzer combined with the photodetector. It can be seen from Figure 6a that the RF output spectrum of the mode-locking fiber laser was obtained, which was located at the fundamental frequency of 21.22 MHz, with a bandwidth of 22 MHz, as well as a resolution of 1 kHz. Moreover, the signal-to-noise ratio (SNR) was about 48 dB. Therefore, the results as shown in Figure 6a suggested that the mode-locking operation exhibited an excellent stability. Additionally, RF spectra with a broad band of 800 MHz were also recorded and are depicted in Figure 6b. As shown, the resolution of the RF spectra was 3 kHz, which further demonstrated that the state of the single mode-locking pulse exhibited a high stability. However, as illustrated in Figure 6b, the top of the RF spectra was not very flat, which shows that the mode-locking operation could be further improved in our work. In addition, the thermal effect of the PVA under high pump power also affects the stability of the mode-locked operation. Due to the high threshold power of the mode-locking operation as mentioned above, the ZrSe_2_–PVA film suffered from thermal effects, which had an impact on the stable mode-locking state. Therefore, the RF spectra within a wide bandwidth shows a component of instability. Based on this, in our future works, we will be focusing on obtaining a more stable mode-locked operation under a lower pump power.

**Figure 7:**
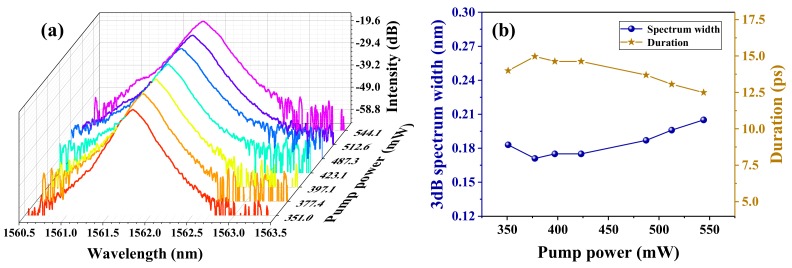
(**a**) The output optical spectrum at different pump powers. (**b**) The 3 dB spectrum width and the theoretical duration of the single mode-locking pulse versus the pump power.

In order to further test the performance of the fiber laser in our experiment, we studied a series of output optical spectra and 3 dB spectrum widths, as well as the theoretical duration of the single pulse at different pump powers. As shown in Figure 7a, when the PCs were fixed in a state of mode-locked operation, the emission spectra of different pump powers were recorded. It is obvious that the intensity and central wavelength of the optical spectra remain almost unchanged when the pump power increased from 351 to 544.1 mW. The corresponding 3 dB spectrum band width and the theoretical monopulse duration are depicted in Figure 7b. When the pump powers were adjusted from the threshold power to the full pump, the 3 dB bandwidth increased from 0.183 to 0.205 nm, and the variety of spectrum width was about 0.034 nm. Meanwhile, the theoretical duration of the single mode-locking pulse, which was calculated according to Equation (2), decreased from 14 to 12.5 ps as the pump powers were regulated from the threshold power to the full pump. As is shown, the narrowest pulse width of 12.5 ps could be obtained when the pump power was set at 544.1 mW. Obviously, the monopulse duration could be further narrowed by increasing the pump power. Thus, our future work will focus on looking for enhancing the damage threshold of the ZrSe_2_–PVA film-type SA in order to further narrow the monopulse duration of the passively mode-locking fiber lasers.

## 5. Conclusions

In conclusion, ZrSe_2_ was used as a SA to demonstrate a passively mode-locked Er-doped fiber laser for the first time. Moreover, the nonlinear absorption characteristics of the ZrSe_2_–PVA film were investigated in our experiment, where the saturation intensity of the ZrSe_2_–PVA film was 12.72 MW/cm^2^, while the modulation depth was 2.3%. The stable passive mode-locked state with a maximum output power of 11.37 mW, under a pump power of 544.1 mW, was obtained. Meanwhile, the narrowest single pulse width was as short as 12.5 ps at a repetition frequency of 21.22 MHz. All experimental results fully verified that the ZrSe_2_–PVA film-type SA had a high damage threshold and excellent nonlinear absorption characteristics, which suggested that few-layer ZrSe_2_ used as an advanced material would have extensive application prospects within the field of ultra-fast pulse fiber lasers.
